# Association analyses for dopamine receptor gene polymorphisms and weight status in a longitudinal analysis in obese children before and after lifestyle intervention

**DOI:** 10.1186/1471-2431-13-197

**Published:** 2013-11-27

**Authors:** Christian L Roth, Anke Hinney, Ellen A Schur, Clinton T Elfers, Thomas Reinehr

**Affiliations:** 1 Department of Pediatrics, University of Washington, Seattle Children’s Research Institute, 1900 Ninth Ave, Seattle, WA 98101, USA; 2 Department of Child and Adolescent Psychiatry, Universitätsklinikum Essen (AöR), University of Duisburg-Essen, Wickenburgstr, Essen 21, 45147, Germany; 3 Internal Medicine, University of Washington Medical Center, 1959 NE Pacific St, Seattle, WA 98195, USA; 4 Pediatric Endocrinology, Diabetes, and Nutrition Medicine, Vestische Hospital for Children and Adolescents Datteln, University of Witten/Herdecke, Dr. F. Steiner Str. 5, Datteln 45711, Germany

**Keywords:** Dopamine receptor polymorphisms, Obesity, Lifestyle intervention, Weight reduction

## Abstract

**Background:**

Dopamine receptors are involved in midbrain reward circuit activation. Polymorphisms in two dopamine receptor genes, *DRD2* and *DRD4,* have been associated with altered perception of food reward and weight gain. The objective of this study was to determine whether the same risk alleles were associated with overweight/obesity and with lower reduction of overweight after a 1-year lifestyle intervention.

**Methods:**

In a longitudinal study the association of polymorphisms in *DRD2* (rs18000497, risk allele: T, formerly A1 allele at the TaqI A1 polymorphism) and *DRD4* (variable number of tandem repeats (VNTR); 48 bp repeat in exon III; risk alleles: 7 repeats or longer: 7R+) was tested on weight loss success following a 1-year lifestyle childhood obesity intervention (OBELDICKS). An additional exploratory cross-sectional case-control study was performed to compare the same *DRD* polymorphisms in these overweight/obese children and adolescents versus lean adult controls. Subjects were 423 obese and 28 overweight children participating in lifestyle intervention (203 males), age median 12.0 (interquartile range 10.0–13.7) years, body mass index - standard deviation score (BMI-SDS) 2.4 ± 0.5; 583 lean adults (232 males); age median 25.3 (interquartile range 22.5–26.8) years, BMI 19.1 ± 1.9 kg/m^2^. BMI, BMI-SDS and skinfold thickness measures were assessed at baseline and after 1 year; genotyping was performed for *DRD2* risk variant rs1800497 and *DRD4* exon III VNTR.

**Results:**

The *DRD2* genotype had a nominal effect on success in the weight loss intervention. The weakest BMI-SDS reduction was in children homozygous for two rs1800497 T-alleles (n = 11) compared to the combined group with zero (n = 308) or one (n = 132) rs1800497 T-allele (-0.08 ± 0.36 vs. -0.28 ± 0.34; p < 0.05). There was no association between the *DRD4* VNTR alleles and genotypes and success in the weight loss intervention. No associations of the risk alleles of the *DRD2* and *DRD4* polymorphisms and obesity were observed in the cross-sectional part of the study.

**Conclusions:**

We did not find association between polymorphisms in *DRD2* and *DRD4* genes and weight status. However, obese carriers of two *DRD2* rs1800497 T-alleles may be at risk for weak responses to lifestyle interventions aimed at weight reduction.

**Trial registration:**

Obesity intervention program “Obeldicks” is registered at clinicaltrials.gov (NCT00435734).

## Background

Genetic factors are involved in individual body weight variation. Midbrain dopamine circuits may play an important role in both addiction and normal eating behavior as they are involved in reward processing, particularly dopaminergic signaling via dopamine receptors 2 and 4 (DRD2, DRD4) [1-3].

Dopamine signaling plays a critical role in the striatum, a brain area that is critically involved in reward and central satiety signaling [4]. In addition, the nucleus accumbens (NAc) and its dopaminergic input from the ventrotegmental area (VTA) have been implicated in reward-seeking behavior, including enabling motor movement towards a reward [5]. These areas are part of a hunger mediating network that includes areas such as the insula, VTA, NAc and anterior cingulate cortex (ACC), which are more active during hunger and fasting and motivate consumption of calorically-dense foods [4,6-8].

Overweight individuals show increased attention to palatable food and find it more rewarding [9]. It is has been suggested that obese individuals tend to overeat in order to compensate for a weak activation of the meso-limbic reward system in response to food intake [10,11]. This could be a consequence of high fat and high carbohydrate intake. However, it is also possible that altered dopamine signaling is a risk factor for development of obesity and thus being a cause rather than a consequence of obesity. The concept of altered reward sensitivity has also been discussed in the context of binge eating disorders, substance addiction, and impulsivity [1]. Obese individuals may show hypofunctioning of food reward circuitry while resting, but hyperfunctioning when exposed to food or food cues [12,13]. However, the role of dopamine, a primary component of reward pathways, in obesity is still controversial [14-16].

Evidence suggests that dopamine-related genes moderate reward circuitry in anticipation or response to food intake. The most commonly tested and referred to *DRD2* polymorphism is rs1800497 (the risk allele T is also known as the *Taq*I A1 allele), which was later shown to lie within the adjacent ankyrin repeat and kinase domain containing 1 gene (*ANKK1*) [17]. In humans a low DRD2 density is associated with the rs1800497 T-allele [18], putatively making individuals less sensitive to the activation of dopamine-based reward circuitry and rendering them more likely to overeat. In fact, binge eating has been shown to be more frequent among obese adults who were homo- or heterozygous for the T allele at rs1800497 [19].

Additional evidence implicates DRD4 signaling in reward sensitivity. DRD4 is a postsynaptic receptor that is principally inhibitory of the second messenger adenylate cyclase. DRD4s are predominantly localized in areas that are innervated by mesocortical projections from the ventral tegmental area, including the prefrontal cortex, cingulate gyrus, and insula [20]. The *DRD4* exon III variable number tandem repeat “7 repeats or longer” allele (*DRD4* 7R+) has been linked to deficient dopamine functioning [20,21].

In functional neuroimaging studies Stice et al. showed that blunted post-meal dorsal striatal activation in carriers of at least one *DRD2* rs1800497 T or *DRD4* 7R + allele(s) was associated with stronger body mass index (BMI) increase in future [9,22]. Therefore we focused on these two variants in children. The question is whether gene variants of dopamine receptors moderate treatment responses and predict success in an obesity intervention based on behavioral modification. There are no studies in children investigating the effect of dopamine receptor risk alleles on outcomes of obesity intervention.

In this study, we genotyped *DRD2* rs1800497 and *DRD4* variable number of tandem repeats (VNTR) in overweight and obese children who underwent a lifestyle intervention, as well as in a lean adult control group. We hypothesized, that the presence of *DRD2* rs1800497 T and/or *DRD4* 7R + alleles are more frequent among overweight/obese vs. lean subjects and are associated with weaker reduction of overweight after a 1 year childhood obesity intervention.

## Methods

### Study groups

Study group 1 (cases) comprised 28 overweight and 423 obese children (see Table 1; 203 males, age median 12.0 y, interquartile range 10.0 – 13.7 y, for all 451 studied children), who participated in a structured lifestyle intervention program (Obeldicks). These children were examined at the outpatient obesity referral centers in Datteln, Germany. Children with syndromal obesity, diabetes mellitus or other endocrine or psychiatric disorders were excluded from the study. Study group 2 (controls) comprised 583 German normal and underweight healthy young adult controls (see Table 1; 231 males; age median 25.3, interquartile range 22.5 – 26.8 y, for details see [23]). Their median BMI was 18.6 (interquartile range 17.7 – 20.6) kg/m^2^. The study was approved by the institutional ethics committees of the Universities Witten/Herdecke and Duisburg-Essen. Written informed consent was obtained from all children and, in case of minors, their parents in accordance with institutional guidelines and with the Declaration of Helsinki.

**Table 1:** **Association of *DRD2/ANKK1 *** **rs1800497 genotypes to baseline parameters and outcomes of a weight loss intervention among overweight/obese children (N = 451)**

	**CC (A2/A2)**	**CT (A1/A2)**	**TT (A1/ A1)**	**CC&CT**	**Additive**^**a**^	**Recessive (T)**	**Dominant (T)**
**N**	**308**	**132**	**11**	**440**			
**Baseline BMI**^**b**^	27.45 ± 4.49	27.19 ± 4.42	26.49 ± 2.45	27.38 ± 4.46	0.686	0.197	0.561
**Change in BMI**^**b,e**^	-0.41 ± 1.95	-0.99 ± 1.93	0.75 ± 2.51	-0.58 ± 1.96	**0.002**	**0.023**	**0.024**
**Baseline BMI-SDS**^**c**^	2.37 ± 0.50	2.35 ± 0.48	2.13 ± 0.38	2.36 ± 0.50	0.285	0.125	0.460
**Change in BMI-SDS**^**c,e**^	-0.26 ± 0.34	-0.34 ± 0.33	-0.08 ± 0.36	-0.28 ± 0.34	**0.015**	0.060	0.090
**Baseline triceps skinfold (mm)**^**b,d**^	31.29 ± 8.80	31.28 ± 11.22	32.05 ± 6.25	31.29 ± 9.55	0.966	0.932	0.850
**Change in triceps skinfold (mm)**^**b,d**^	-2.40 ± 10.41	-5.38 ± 11.59	-1.86 ± 6.47	-3.27 ± 10.83	0.053	0.639	0.027
**Baseline subscapular skinfold (mm)**^**b,d**^	30.12 ± 9.75	29.53 ± 11.38	30.82 ± 5.95	29.95 ± 10.25	0.837	0.873	0.741
**Change in subscapular skinfold (mm)**^**b,d**^	-2.71 ± 11.05	-3.24 ± 10.67	2.91 ± 7.67	-2.87 ± 10.92	0.204	0.086	0.952

All values are mean ± SD. After adjustment for multiple comparisons P-values <0.025 were considered as significant (in bold letters). ^a^Additive (overall) p-value for the model comparing CC, CT, TT.

^b^Linear Regression P-value adjusted for age, puberty and gender. ^c^Unadjusted linear regression P-value. ^d^Missing values 1-4%; ^e^Missing values 5-22%.

### Anthropometric data and obesity related measures

Body weight of patients and controls was evaluated using the following BMI calculation: BMI = weight [kg]/ height^2^ [m^2^]. In children this was expressed as a standard deviation score (BMI-SDS) (see statistical methods). Overweight and obesity were defined according to the International Task Force of Obesity by BMI-SDS between the 90^th^ and 97^th^ percentile and above the 97^th^ percentile, respectively, according to age and gender using population specific data. Height was measured to the nearest centimeter using a rigid stadiometer. Weight was measured in underwear to the nearest 0.1 kg using a calibrated balance scale. Height-SDS, weight-SDS and BMI-SDS were calculated according to German percentiles as mentioned in a previous study [24]. Pubertal developmental stage was assessed using the standards from Marshall and Tanner. Triceps and subscapularis skinfold thicknesses were measured in duplicate using a caliper and averaged [25].

### Obesity intervention

As part of the study, all 451 children who were treated at the Vestische Kinderklinik, Datteln, participated in the 1-year German obesity intervention program “Obeldicks” which has been described previously in more detail [26] and is registered at clinicaltrials.gov (NCT00435734). Briefly, the 1-year intervention program is based on physical exercise, nutrition education, and behavioral therapy, including the individual psychological care of the child and his or her family [26]. The exercise therapy took place once per week throughout the whole intervention year.

### Dopamine receptor gene variants

Blood samples were provided from all participants to extract DNA using a standard salting-out method. We genotyped the *DRD2* single nucleotide polymorphism (SNP) rs1800947 as described previously [9,22]. Genotyping was performed by PCR (298 bp amplicon using the primers: forward 5′-GGCTGGCCAAGTTGTCTAAA, reverse 5′-CCTGAGTGTCATCAACCTCCT) and subsequent digest by *Taq*I; detailed conditions for the PCR-RFLP can be obtained by the authors. The *DRD4* exon III VNTR was genotyped as we described previously [27]. Genotypes of 82 of the underweight controls had been used for our previously published association study [27].

### Statistical analysis

Means and standard deviations were calculated for all measures, stratified by genotype. The first analysis separately examined the relationship of *DRD2* rs1800497 and *DRD4* VNTR to BMI in all adult and child subjects. *DRD2* rs1800497 genotypes were CC, CT or TT. A combined group (CC and CT) was compared to subjects who were homozygous for the rs1800497 T (risk) allele. *DRD4* exon III VNTR polymorphism was classified as having no 7R+, one 7R + or two 7R + alleles . The second analysis tested obesity intervention outcomes in obese children in relation to *DRD2* and *DRD4* genotypes. Longitudinal changes in BMI-SDS over the course of the 1 year “Obeldicks” program were evaluated. The rationale for testing an *additive genetic model* was to test the effect of zero vs. one vs. two minor alleles on BMI status and obesity intervention outcomes, which is usually the best choice if the true genetic model is not known [28]. In addition, we tested the *dominant model* under the assumption that one risk allele is sufficient for development of obesity and to affect obesity intervention outcomes [9,22]. As the genetic model is not well established for the studied variants, we finally also investigated whether two risk alleles are necessary to have an impact on BMI status and intervention outcomes in a *recessive model* (homozygous for the risk allele versus all other genotypes). Due to the varying distribution of BMI over different stages of childhood, the LMS method was utilized to calculate BMI-SDS as a normalized measurement for the degree of overweight. The LMS method was chosen because it summarizes the data in terms of three smooth age-specific curves called L (λ), M (μ), and S (σ), based on German population-specific data [24,29]. The M and S curves correspond to the median and coefficients of variation (CVs) of BMI for German children at each age and gender, whereas the L curve allows for the substantial age-dependent skewness in the distribution of BMI. The assumption underlying the LMS method is that after Box-Cox power transformation, the data at each age are normally distributed [29]. We investigated the effect of the genotypes on anthropomorphic measurements both at baseline and the changes during weight intervention. Linear regression analyses were performed using Stata 12 software (Stata Corp, College Station, TX) and were calculated both unadjusted and adjusted for gender, age, puberty status and BMI-SDS as applicable. No. of risk alleles, gender, and puberty status were treated as nominal variables for all analyses. Overall effects were tested and indicator variables were used to assess the associations between risk and non-risk genotypes.

Student’s t-tests were performed using Prism 5 software (GraphPad, La Jolla, CA) for two group comparisons of measurements between combined zero or one rs1800497 T vs. non-rs1800497 T alleles. All reported p-values in tables are two-sided, nominal, and are adjusted by Bonferroni correction [28] for multiple testing (2 tests: BMI status, skinfold thickness) and to confounders if stated. The consistency of genotype frequencies was tested with Hardy Weinberg equilibrium. Pearson’s chi squared tests were performed using Stata 12 software (Stata Corp, College Station, TX) for comparison of *DRD2* rs1800497 T allele and *DRD4* 7R + allele and genotype distributions between children and lean adult controls.

## Results

**Figure 1:**
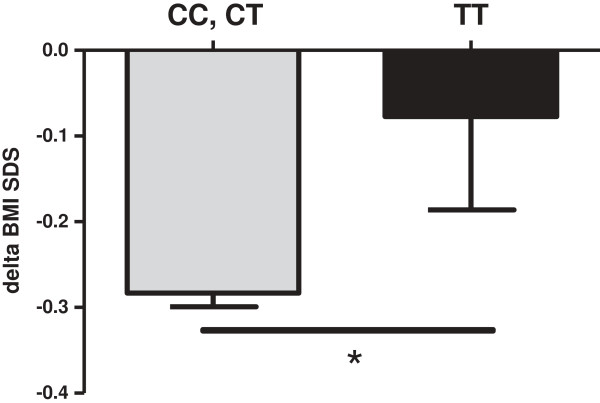
**Change of BMI-SDS after 1 year lifestyle intervention in 451 overweight/obese children.** *p = 0.046 homozygous TT risk allele status vs. CC and CT combined by students t-test.

In longitudinal data analyses of treatment outcomes, there was an overall effect of *DRD2* genotype on weight loss success (Table 1). The strongest BMI and BMI-SDS reductions occurred among children with the *DRD2* CT genotype. The intervention had a weak or no effect among children with TT genotypes as compared to children with no or one rs1800497 T allele (CC, CT) (Table 1, Figure 1). Of the 11 probands homozygous for the T allele at rs1800497, 6 were in the quartile of the weakest BMI z-score reduction (Fisher’s exact test across quartiles p = 0.154, Table 2). There was a trend in changes of subscapular skinfold thickness showing no reduction in TT vs. reduction in CC and CT (Table 1).

**Table 2:** Delta BMI z-score changes in quartiles vs. rs1800497T allele status, n = 440 obese children participating in lifestyle intervention

**Quartile:**	**1**	**2**	**3**	**4**
Delta BMI z-score	-0.72 ± 0.24	-0.36 ± 0.06	-0.15 ± 0.07	0.12 ± 0.13
CC N(%)	70(23.2)	73(24.2)	82(27.2)	77(25.5)
CT N(%)	39(30.7)	35(27.6)	26(20.5)	27(21.3)
TT N(%)	1(9.1)	2(18.2)	2(18.2)	6(54.5)

Fisher’s exact test p = 0.154.

We detected no association of *DRD4* VNTR alleles or genotypes on BMI, BMI-SDS or skinfold thickness at baseline. Nor were differences present in longitudinal changes in these parameters among the *DRD4* 7R + allele groups (Table 3).

**Table 3:** **Association of *DRD4 *** **exon III variable number of tandems repeat genotypes to baseline parameters and outcomes of a weight loss intervention in 451 overweight/obese children**

	**No 7R + alleles**	**One 7R + allele**	**Two 7R + alleles**	**Additive**^**a**^	**Recessive**	**Dominant**
**N**	**285**	**148**	**18**			
**Baseline BMI**^**b**^	27.25 ± 4.39	27.41 ± 4.58	28.97 ± 3.81	0.279	0.355	0.456
**Change in BMI**^**b,d**^	-0.48 ± 1.95	-0.62 ± 2.00	-1.01 ± 2.42	0.479	0.434	0.362
**Baseline BMI SDS**^**c**^	2.34 ± 0.48	2.38 ± 0.53	2.5 ± 0.3	0.413	0.336	0.253
**Change in BMI SDS**^**c,d**^	-0.25 ± 0.32	-0.32 ± 0.36	-0.30 ± 0.43	0.358	0.746	0.152
**Baseline triceps skinfold (mm)**^**b,e**^	31.17 ± 9.45	30.63 ± 7.08	34.82 ± 5.11	0.167	0.107	0.825
**Change in triceps skinfold (mm)**^**b,e**^	-2.94 ± 11.29	-3.03 ± 6.45	-4.03 ± 6.71	0.915	0.867	0.976
**Baseline subscapular skinfold (mm)**^**b,e**^	29.95 ± 10.58	29.31 ± 8.29	32.45 ± 5.56	0.440	0.344	0.776
**Change in subscapular skinfold (mm)**^**b,e**^	-2.73 ± 11.09	-2.04 ± 9.15	-3.27 ± 9.74	0.805	0.935	0.573

All values are mean ± SD. ^a^Additive (overall) p-value for the model comparing 0, 1, or >1 repeats.

^b^Linear Regression P-value adjusted for age, puberty and gender. ^c^Unadjusted linear regression P-value. ^d^Missing values 1-4%; ^e^Missing values 5-20%.

In the additional case control study, risk allele distribution was compared between obese children and lean controls and there was no difference in the proportion of subjects with one (CT), two (TT), or no (CC) T alleles at rs1800497 (p-value = 0.840, χ^2^ = 0.348; Pearson’s Chi-squared test, see values in Table 1). Similarly, the distribution of zero, one, or two risk alleles of *DRD4* 7R + was not different between the obese children vs. lean controls (p-value = 0.728; χ^2^ = 0.636; Pearson’s Chi-squared test) (Table 4).

**Table 4:** **Distribution of *DRD2/ANKK1 *** **rs1800497 alleles and *DRD4 *** **exon III variable number of tandems repeat alleles in relation to BMI among all adult and pediatric subjects**

	**Adults (lean)**				**Children (overweight or obese)**				
	**N (% of total) n = 583**	**Age**	**Sex**	**BMI**	**N (% of total) n = 451**	**Age**	**Sex**	**BMI**	**BMI-SDS**
**rs1800497**									
**CC (A2/A2)**	407 (69.8)	25.4 ± 4.5	161 M/246 F	19.2 ± 2.0	308 (68.3)	10.8 ± 2.6	139 M/169 F	27.5 ± 4.5	2.4 ± 0.5
**CT (A1/A2)**	161 (27.6)	25.1 ± 4.3	64 M/97 F	19.1 ± 1.9	132 (29.3)	10.7 ± 2.7	60 M/72 F	27.2 ± 4.4	2.3 ± 0.5
**TT (A1/A1)**	15 (2.6)	24.4 ± 3.0	6 M/9 F	18.2 ± 1.1	11 (2.4)	11.3 ± 1.7	4 M/7 F	26.5 ± 2.5	2.1 ± 0.4
**DRD4 7R+**									
**No**	357 (61.2)	25.6 ± 4.6	149 M/208 F	19.2 ± 2.0	285 (63.2)	10.8 ± 2.6	135 M/150 F	27.2 ± 4.4	2.3 ± 0.5
**One**	198 (34.0)	24.8 ± 4.1	71 M/127 F	19.0 ± 1.9	148 (32.8)	10.8 ± 2.7	62 M/86 F	27.4 ± 4.6	2.4 ± 0.5
**Two**	28 (4.8)	25.4 ± 4.0	11 M/17 F	18.8 ± 1.5	18 (4.0)	11.4 ± 2.1	6 M/12 F	29.0 ± 3.8	2.5 ± 0.3

Age and BMI values are Mean ± SD.

## Discussion

The current study examined associations between a *DRD2* and a *DRD4* polymorphism and weight loss during a lifestyle intervention. There was an overall effect of *DRD2* genotype on BMI reduction in the lifestyle intervention. Homozygotes for the rs1800497 T allele showed a lower weight status reduction in response to lifestyle intervention than carriers of the other genotypes. There was no association of the *DRD4* VNTR polymorphism with the analyzed phenotypes. This is the first report on the association of dopamine receptor variant status and childhood obesity intervention outcomes. However, in the additional cross-sectional part of the study, we did not find association for either the *DRD2* or the *DRD4* polymorphism alleles or genotypes and overweight or obesity.

We postulated that both *DRD* gene polymorphisms evoke excessive calorie consumption, which may reflect overall impaired dopamine-driven response inhibition leading to obesity and poor obesity intervention outcomes [30]. Response inhibition refers to the neural process by which unnecessary or inappropriate motor action is suppressed [31-35]. Impaired response inhibition is a behavioral trait of which impaired satiety may be one manifestation. A related trait – impulsivity – has been linked to obesity [36-38] and poor obesity treatment outcomes in children [37].

In the longitudinal part of the study, gene polymorphisms in *DRD2* did predict (nominal p-value < 0.05) outcomes in the lifestyle intervention. Carriers of two *DRD2* rs1800497 T alleles may be at risk for weaker weight status reduction in response to lifestyle intervention. This group seems to be enriched in lowest quartile for BMI z-score reduction (Table 2). However, these results need to be regarded with caution as they did not reach statistical significance upon Bonferroni correction. Thus, even though the number of children in this group was a small proportion of the total children enrolled, children with the TT genotype may represent a larger proportion of children who do not do well in lifestyle interventions. We did not find evidence that carriers of one rs1800497 T allele are at risk for obesity or reduced success during obesity intervention which needs to be discussed in context with prior results of functional neuroimaging studies by Stice et al. in which the presence of one risk allele was sufficient to modulate the relation between food reward and future weight gain [9,22]. Although the authors reported that the rs1800497 T (A1) allele status did not predict increase in BMI over follow-up, they found that the rs1800497 T allele moderated the relations of brain responses during exposure to appetizing vs. unappetizing food to risk for increases in BMI over the 1-year follow-up. Therefore, it is possible that the effects of *DRD* variant status on neuronal activation is stronger than on weight status per se, as individuals in our study were seeking weight loss and therefore may already have compensated somewhat for this predisposition. Moreover, our data support the hypothesis that children with a single risk allele may actually be particularly responsive to lifestyle intervention as they demonstrated significantly greater reductions in BMI. Behavioral therapy and nutrition education might be sufficient to engage cognitive control and counteract predispositions in this population, which, if our findings are replicated, would be encouraging.

Humans who are homo- or heterozygous for *DRD4* 7R + alleles have shown higher peak body mass in cohorts at risk for obesity [39,40], greater food cravings [41], as well as smoking, alcohol, and drug cravings [42-44]. We did not find association for *DRD4* 7R + allele carriers to obesity, or weight loss success in a childhood obesity lifestyle intervention. In addition, there are also no published studies showing an association between *DRD4* 7R + alleles and weight status or responses to obesity intervention in this age group. Potentially this is not a predominating factor for weight status and response to obesity intervention in the age group of our studied children.

Studying children is advantageous as the obesity is not yet chronic and exposure to a calorie dense diet was not very long. Longer exposure has been hypothesized to reduce dopamine signaling via receptor down-regulation. In the additional cross-sectional part of the study, we did not find evidence that the risk alleles at the tested *DRD2* and *DRD4* polymorphisms are associated with measures of obesity. These data are not inconsistent with prior findings, as the *DRD2* rs1800497 T allele was associated with increased body mass in some studies [45-47], while other studies do not show association [48,49]. In recent a longitudinal study investigating the association between change in BMI from adolescence to young adulthood and polymorphisms in genes involved in serotonergic and dopaminergic functioning, no significant associations were found between *DRD2* rs1800497 T allele or *DRD4* 7R + allele and BMI categories [50]. However, a polymorphism in the monoamine oxidase A (MAOA) gene, that encodes an enzyme that metabolizes dopamine, serotonin and noradrenaline, was associated with increased BMI which further supports that the gene variants involved in dopamine metabolism might have an impact on body weight change.

Strengths of this study include the relatively large sample size for the childhood obesity intervention and the longitudinal study design. However, limitations persist that should be discussed. First, adiposity was assessed by indirect estimations (BMI, BMI-SDS; skinfold thickness) [51]. Second, we analyzed the effects of the *DRD* gene polymorphisms only on anthropometric measures and were not able to include any behavioral tests or data on eating. Future studies should include assessment of eating behaviors. Third, in the exploratory cross-sectional part of our study, the lean control group consisted of young adults. Although obese children and adolescents frequently become obese adults [52] and lean adults were most likely lean children, it is possible that some of the lean adult controls were obese during childhood. However, we deem lean adults as better controls for association studies than lean children, as a proportion of the lean children might become obese adults. Hence, lean children might harbor ‘obesity alleles’ and therefore decrease the power of the association study. Finally, we investigated the effect of two *DRD* polymorphisms in our study, but other *DRD* polymorphisms could have an impact as well [3,50,53].

## Conclusions

Our findings contribute to a further understanding of the relation between alterations in dopamine receptor structure and/or function that have previously been shown to lead to compromised dopamine signaling in reward brain areas and higher risk for developing obesity. Although we did not demonstrate an association between *DRD4* VNTR and weight status, we found that carriers of *DRD2* rs1800497 T alleles are at risk for weak responses to lifestyle interventions aimed at weight reduction.

## Abbreviations

ACC: Anterior cingulate cortex; ANKK1: Ankyrin repeat and kinase domain containing 1; BMI: Body mass index; BMI-SDS: Body mass index – standard deviation score; CVs: Coefficients of variation; DRD2: Dopamine receptor 2; DRD4: Dopamine receptor 4; NAc: Nucleus accumbens; VNTR: Variable number of tandem repeats; VTA: Ventrotegmental area.

## Competing interests

The authors declare that they have no competing interests.

## Authors’ contributions

AH, TR, and CR developed the study design. CE, ES, TR, and CR performed statistical analyses. TR performed and supervised anthropometrical measurements. AH supervised the genetic tests. CR wrote the first draft of the paper. All authors discussed the findings. All authors read and approved the final manuscript.

## Pre-publication history

The pre-publication history for this paper can be accessed here:

http://www.biomedcentral.com/1471-2431/13/197/prepub
